# Removal

**Published:** 1881-12

**Authors:** 


### REMOVAL.

The Editorial Office of the Dental Register has been removed from 117 West Fourth Street, to No. 122 West Seventh Street.

Also, the Office of J. Taft and William Taft has been removed to the same place.

